# Portable direct spraying porous nanofibrous membranes stent-loaded polymyxin B for treating diabetic wounds with difficult-to-heal gram-negative bacterial infections

**DOI:** 10.1016/j.mtbio.2024.101365

**Published:** 2024-11-24

**Authors:** Xiaolan Ou, Wenlai Guo, Heng Tian, Daojiang Yu, Rui Li, Guanghui Gao, Wenrui Qu

**Affiliations:** a Department of Hand Surgery, The Second Hospital of Jilin University, Changchun, Jilin Province, 130041, China; b Department of Plastic Surgery, Nuclear Industry 416 Hospital, the Second Affiliated Hospital of Chengdu Medical College, Chengdu, 610051, China; c Polymeric and Soft Materials Laboratory, Advanced Institute of Materials Science, School of Chemical Engineering, Changchun University of Technology, No. 2055, Yan'an Street, Changchun, 130012, China; d Joint International Research Laboratory of Ageing Active Strategy and Bionic Health in Northeast Asia of Ministry of Education, Jilin University, Changchun, Jilin Province, 130041, China

**Keywords:** Infected diabetic wounds, Electrospinning, Macrophage polarization, Immunoregulation, Neovascularization

## Abstract

Gram-negative bacteria infections in diabetic wounds are complicated to control, leading to amputation and even death in severe cases. There is an urgent need to develop effective therapeutic strategies. In recent years, electrospinning has attracted much attention due to its resemblance to extracellular matrix (ECM), which can regulate local cellular proliferation, migration, differentiation, etc.; however, its use is limited by its high cost and difficulty in transportation. This study proposes a portable direct-injection porous fibre scaffold containing polymyxin B (PMB) for local slow release for treating diabetic wounds infected with difficult-to-heal Gram-negative bacteria. The handheld portable electrospinner is lightweight and easy to operate and can be directly sprayed in situ to cover wounds with irregular shapes and sizes. When covering the wound in situ, the PVB/PVP nanofiber membrane can protect it from the external environment. Meanwhile, the nanofiber membrane dressing has a porosity of 20 % and a controlled drug-loading capacity. What's more, the evaluation of a whole skin defect model of type II diabetes mellitus infected with Gram-negative bacteria showed that the PMB-loaded nanofiber membrane could effectively inhibit Gram-negative bacteria infection, promote collagen deposition and re-epithelialization, and regulate the polarization of M1-type macrophages to M2-type macrophages, thereby controlling inflammation and promoting vascular regeneration, and significantly accelerating the healing of diabetic wounds. Overall, portable direct-injection porous fibre scaffold-loaded drugs are essential for healing difficult-to-heal wounds as local slow-release drug delivery.

## Graphical abstract

Schematic images of the in-situ direct spraying porous electrospinning process of PVB/PVP-PMB and the mechanisms of accelerating diabetic wound healing in gram-negative bacterial infected diabetic wound of nanofibrous membranes.

**Figure:**
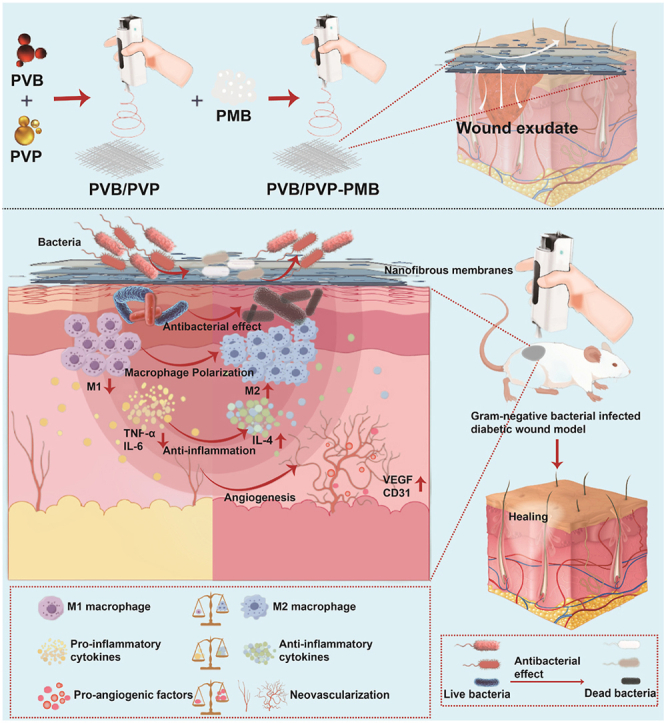

## 1 Introduction

Currently, chronic diabetic wounds have become a severe global burden due to high morbidity, mortality, and healthcare costs [[1], [2], [3]]. Diabetic wounds typically have a complex wound microenvironment of impaired local immunomodulation (Polarization of M1-type macrophages to M2-type macrophages), persistent chronic inflammation, and impaired angiogenesis [4,5]. This microenvironment makes diabetic wounds more susceptible to infection than other types of wounds, leading to increased inflammation and deep tissue damage, further delaying the healing of chronic diabetic wounds. Meanwhile, the incidence of diabetic wounds infected by Gram-negative bacteria is growing with the misuse of antibiotics and the tendency of Gram-negative bacteria to be found in immunocompromised patients [6,7]. However, because the outer membrane of Gram-negative bacteria is usually impermeable and inherently resistant, and because bacteria can easily modify it to become more resistant to acquired and adaptive resistance, treating infections caused by them is highly challenging [[8], [9], [10]]. The resistance level is rapidly evolving along with the over-abuse of antimicrobial agents. This leaves the clinician facing a drugless situation. Therefore, in the fight against quickly emerging bacterial resistance, we cannot rely exclusively on discovering new antibiotics; we must also find rational ways to apply older antibiotics. An example is polymyxin B (PMB). Due to its unique mechanism of action, polymyxin B has been increasingly used as a “last-line” treatment for treating Gram-negative bacterial infection [8,11]. However, polymyxin B has been abandoned due to its susceptibility to severe nephrotoxicity and neurotoxicity [12,13]. But, in the absence of new and more effective drug therapy, a rational route for utilizing polymyxin B drugs should be preferred.

Several bioactive wound dressings have been developed to carry antibiotics for infection control in recent years, such as hydrogels and scaffolds (3D-printed scaffolds and nanofiber scaffolds). Wound dressings based on nanofibrous scaffolds are interesting due to their unique physical and structural properties. For example, nanofibrous scaffolds exhibit an extracellular matrix (ECM)-like structure, which can mimic the natural tissue microenvironment to promote tissue regeneration and provide structural support for cell migration, proliferation, adhesion, and differentiation [[14], [15], [16]]. Moreover, nanofibrous membranes' high porosity and specific surface area allow oxygen permeation, absorption of wound exudates, and protection from dehydration, creating an excellent wound-healing microenvironment [17,18]. However, despite this potential, conventional electrostatic spinning techniques require that the nanofiber membrane be first prepared and then used as a wound dressing to cover the wound [19,20]. As a result, traditional electrospinning silk dressings are usually fixed in shape and size, so they cannot be used for irregular wounds. This dramatically reduces the practical application of electrospinning technology [19]. In this study, a handheld portable electrostatic spinning machine was used. Using this device, nanofibrous membranes can be generated by direct spraying in situ in wounds, which can completely cover any irregularly shaped and sized wounds, bringing hope for the practical application of electrostatically spun nanofibrous scaffolds in our clinical work and even in our daily life.

Currently, a variety of materials can be used for electrostatic spinning, such as poly (-lactic acid) (PLA) [21], poly (-glycolic acid) (PGA), polyvinyl pyrrolidone (PVP) [22], polycaprolactone (PCL) [22], and polyvinyl alcohol (PVA), etc [23]. However, there are few qualified electrostatic spinning materials. In this study, we chose ethanol-soluble polyvinyl butyral (PVB) [24], which is biocompatible, non-toxic but also hydrophobic, and insoluble in water. A small amount of hydrophilic polyvinyl pyrrolidone (PVP), which has good solubility in ethanol and makes the prepared nanofibrous membrane hydrophilic, was added. However, the structure of the nanofibrous membrane would not dissolve due to the presence of PVB. Therefore, PVB/PVP nanofiber membrane is non-toxic and harmless to human tissues and plays a protective role in isolating wounds from the external environment, providing conditions for in-situ electrostatic spinning. In addition, since polymyxin B is insoluble in ethanol, we used the suspension electrostatic spinning method, i.e., polymyxin B was homogeneously dispersed in PVB/PVP solution, and then electrostatic spinning was performed at the wound site to cover the diabetic wound in situ.

In conclusion, in this study, a direct spraying porous nanofiber scaffold was prepared by a portable handheld electrostatic spinning device using suspension electrostatic spinning technology to cover diabetic wounds in situ. PVB, as a fibre support skeleton, and PVP, as a polymer surfactant, in situ coverage of the wound can play a protective role in isolating the wound from the external environment, providing a good healing environment conducive to wound healing. The porous nanofiber scaffolds were equipped with polymyxin B, which could not only effectively control Gram-negative bacterial infections in diabetic wounds but also improve the local immunomodulatory ability of diabetic wounds, control inflammation, and promote vascular regeneration, and significantly accelerate the healing of diabetic wounds.

## 2 Results and discussions

### 2.1 Handheld electrospinning machine and fabrication of nanofibrous membranes

**Fig. 1:**
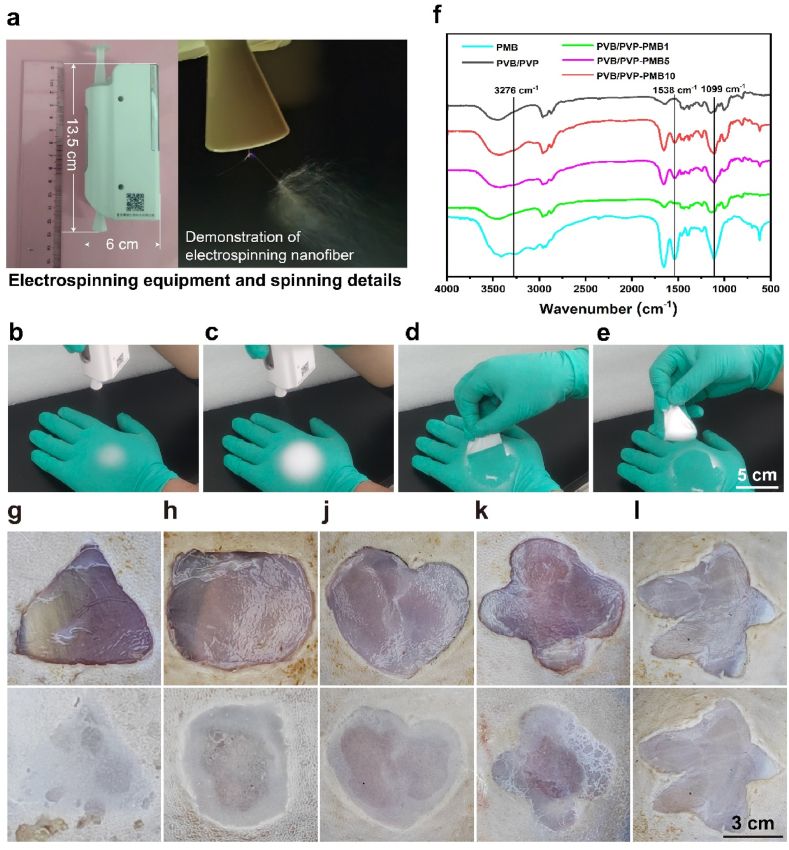
Handheld electrospinning equipment and details of its spinning. (a) Handheld electrospinning apparatus and view of the electrospinning jet from the spout. (b–e) Dynamic process of handheld electrospinning for the preparation of wound dressings. (Scale bar, 5 cm). (g – I) In situ coverage of a wide range of irregular wounds by handheld electrospinning (Scale bar, 3 cm). (f) FTIR spectra of PMB, PVB/PVP, and PVB/PVP-PMB sample.

As shown in Fig. 1a, the handheld electrostatic spinning device is very small, with a length of only 13.5 cm and a width of only 6 cm. However, the results of the nanofibrous dressings prepared by handheld electrostatic spinning were the same as those of the conventional electrostatic spinning machine. The dynamic process and key steps of wound dressing preparation were demonstrated to validate the feasibility of the handheld electrostatic spinning machine for further preparing wound dressings at the wound site (Fig. 1b–e and Movie S1, Supporting Information). As shown in Fig. 1b–c, the PVB/PVP wound dressing is compliant. In addition, it can be easily removed during dressing changes (Fig. 1d–e) and cleared well after contact with tissue fluid (Fig. S1). To further demonstrate that the handheld electrospinning machine can cover irregularly shaped wounds in situ, this study used a handheld electrostatic spinning machine to prepare nanofibrous scaffold dressings directly in situ at the wound site. As shown in Fig. 1g–l, the handheld electrospinning nanofibrous scaffolds can cover irregular wound shapes well in situ with high adaptability and potential clinical applicability.

Supplementary data related to this article can be found online at https://doi.org/10.1016/j.mtbio.2024.101365

The following are the Supplementary data related to this article:

Video. 1 Video. 1

In addition, FT-IR spectra of pure polymyxin B, PVB/PVP, and PVB/PVP-PMB after drug loading are shown in Fig. 1f. The band at 3413^−1^ cm is attributed to the stretching vibration of N-H and the deformation of N-H. The band at 1539 cm^−1^ is attributed to the stretching vibration of the benzene ring on the polymyxin B spectrum, indicating the stretching vibration of C-O associated with CH-OH. After loading polymyxin B on the nanofibrous membrane, polymyxin B vibrations, such as 3413 cm^−1^, 1539 cm^−1^ and 1110 cm^−1^, appeared. These results suggest that the drug may be loaded inside of the fiber or on the surface of fiber.

### 2.2 Properties and characterization of nanofibrous membranes

#### 2.2.1 Morphological characteristics of nanofibrous membranes

**Fig. 2:**
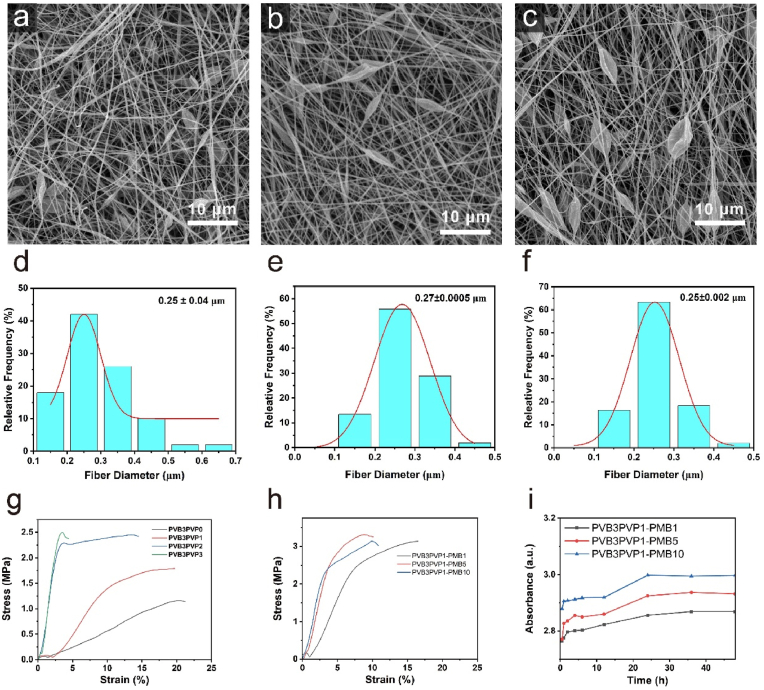
Properties and Characterization of nanofiber membrane. SEM images (a) PVB/PVP-PMB1, (b) PVB/PVP-PMB5, (c) PVB/PVP-PMB10, respectively (n = 3. Scale bar, 10 μm). Fiber diameter distribution of (d)PVB/PVP-PMB1, (e) PVB/PVP-PMB5, (f) PVB/PVP-PMB10, respectively. (g–h) Tensile curves of nanofiber membrane. (i) Sustained release profile of nanofiber membrane.

Fig. 2a–c shows SEM images of PVB/PVP-PMB loaded with different concentrations of the drug polymyxin B. The images suggested that the fibers of PVB/PVP-PMB membrane were long cylindrical strip overall and some fibers had a spindle shape. Fig. 2d–f shows the fiber diameters of membranes PVB/PVP-PMB1, PVB/PVP-PMB5, and PVB/PVP-PMB10, respectively. The drug-loaded concentration had no significant effect on the fibre diameter of the fibrous membrane. However, as the concentration of the drug polymyxin B increased, the number of spindle fibres in the fibrous membrane increased, which may be due to the increase in the amount of PMB-loaded nanofibrous membrane.

#### 2.2.2 Tensile property of nanofibrous membranes

As shown in Fig. 2g, with the increase of PVP content, the tensile strength of the PVB electrostatically spun film increases while the elongation at break decreases; this may be because the addition of PVP enhances the intermolecular forces. This intermolecular interaction enhances the cohesion of the material, making it more difficult for the molecules to slip relative to each other when subjected to tensile forces, thus increasing the tensile strength and decreasing the elongation at break. Further consideration of choosing the fiber membrane of PVP3PVP1 as the drug-carrying matrix ensures that the fiber membrane has a specific elongation at break (19.68 %) along with excellent tensile strength (1.79 MPa). The mechanical strength of the prepared nanofibrous membranes was further improved by adding PMB into the spinning solution (Fig. 2h), which may be attributed to the fact that the drug molecules can be uniformly distributed in the electrostatically spun membranes and play a specific filling role, increasing the denseness and rigidity of the nanofibers.

#### 2.2.3 Water contact angle, porosity, and water vapor transmission rate (WVTR) of nanofibrous membranes

The hydrophilicity and hydrophobicity of PVB/PVP wound dressings were evaluated. As shown in Fig. S2a, the water contact angle decreased significantly with the addition of PVP, indicating an increase in the hydrophilicity of the nanofibrous dressing, mainly due to the hydrophilic nature of PVP. It was shown that increasing the surface hydrophilicity of hydrophobic materials can improve cell adhesion, especially cell growth, and positively contribute to wound healing [25]. However, when the PVB content decreased from 3 % to 1 % and the PVP content was increased from 1 % to 3 %, the water contact angle of PVB/PVP wound dressings show a mild downward trend. To maintain the moderate hydrophobicity and hydrophilicity of the nanofiber membrane, PMB was chosen to be added to the matrix of PVB3/PVP1. After the addition of PMB, the water contact angle of the wound dressings decreased significantly with the increase of PMB content (Fig. S2b), which was mainly due to the hydrophilicity of PMB, and the decrease of water contact angle (WAC) caused by the increase in roughness of the nanofibrous dressings with the increase of PMB content. The WCA of PVB/PVP-PMB1, PVB/PWP-PMB5, and PVB/PVP-PMB10 were 81.5°, 72.5°, and 75°, respectively. The contact angles were all less than 90°, indicating that the PVB/PVP-PMB nanofibrous membranes had good hydrophilicity, which helped the nanofibrous membranes bind more closely to the wound. The tight binding of the electrospinning scaffolds of extracellular matrix-like material to the wound helps to regulate the local microenvironment of the wound. In addition, as shown in Fig. S2c, the pores of the PVB/PVP nanofibrous gradually increased with the increase of PMB content, which also made it easier for fluids to enter the pores between the fibers, thus increasing the contact area between fluids and PMB (Fig. S2c). Nanofibrous scaffolds with a porous structure provide sufficient oxygen and moisture to prevent the wound bed from drying out and contribute to the release of drugs to control bacterial infections effectively. The water vapor permeability of PVB/PVP nanofibrous is also proportional to the fiber porosity, as shown in Fig. S2d. This is because higher porosity results in lower resistance to vapor-liquid exchange. In conclusion, the PVB/PVP-loaded PMB nanofibrous membranes wound dressing has excellent hydrophilicity with moderate porosity and water vapor permeability. It absorbs wound exudate while maintaining a moist environment and provides sufficient oxygen and strong antimicrobial capacity, all which benefit wound repair.

#### 2.2.4 Drug release of nanofibrous membranes

The results of the release profile of PMB in PBS solution at 37 °C are shown in Fig. 2i, PMB exhibited a faster release rate within 0.5 h, which was mainly due to the sudden release of the drug due to diffusion of the drug attached to the surface of the dressing down the concentration gradient. Subsequently, the release rate slowed down due to the dissolution of PVP in the nanofibers, resulting in a slow release of the encapsulated drug. Finally, it stabilized at 36 h, and the drug release was almost complete.

### 2.3 In vitro and in vivo biocompatibility testing

**Fig. 3:**
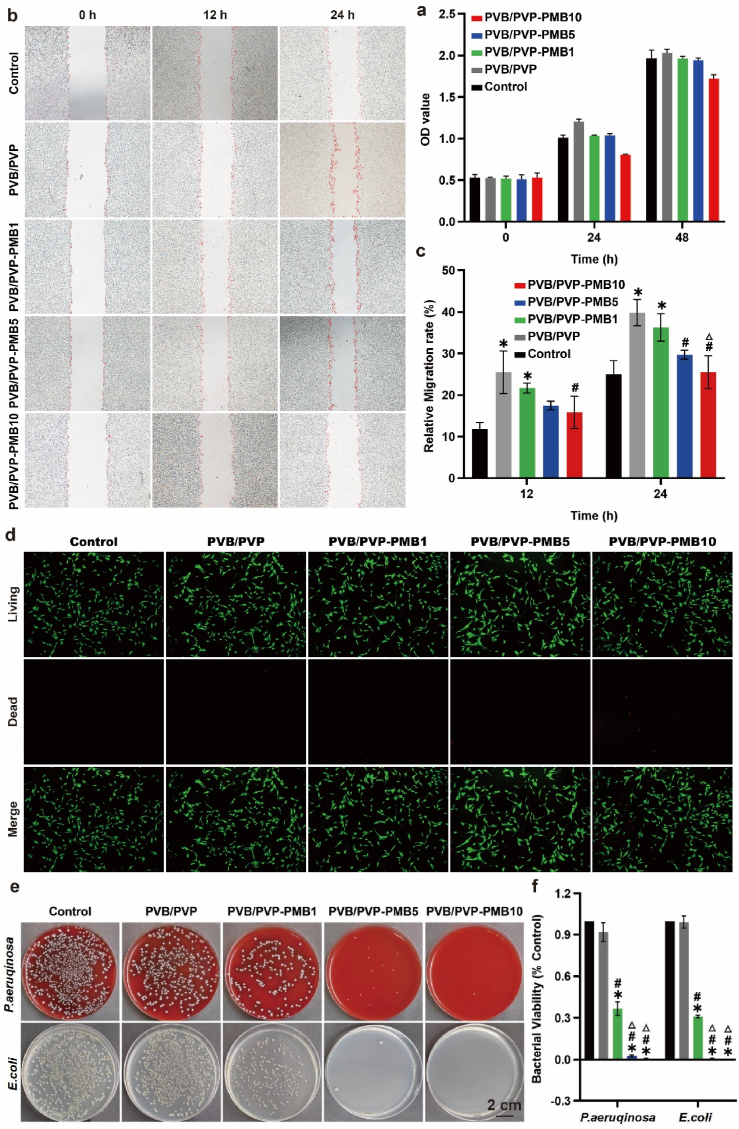
In vitro and in vivo evaluation of nanofibrous membranes' biocompatibility and antimicrobial properties. (a) The OD values of each group are at 0, 24, and 48 h. (b) Representative images of cell scratching experiment. (c) Quantification histogram represented migration rate (n = 3). (d) Fluorescence images of NIH/3T3 cells after live/dead staining on day 1 (Scale bar, 200 μm). (e) Antimicrobial efficacy against *E. coli* and *P. aeruginosa* (Scale bar, 2 cm) and corresponding statistical results (f, n = 3).

The cell proliferation during skin wound healing is mainly related to the proliferation by fibroblasts [26]. In this study, the cytocompatibility of the electrospinning silk scaffold dressing was tested by the proliferation assay of NIH/3T3 fibroblasts. As shown in Fig. 3a, the number of cells in each group gradually increased after 24 and 48 h of exposure to each group of dressings. Among them, the highest number of PVB/PVP, PVB/PVP-PMB1, and PVB/PVP-PMB5 groups reached more than 85 % of the PVB/PVP group, respectively. In contrast, PVB/PVP-PMB10 reached only 80 % of the blank control, indicating that PVB/PVP electrospinning silk dressings could promote cellular proliferation, and low doses of PMB had no significant effect on cellular value-added, with the increase of PMB content, it has certain cytotoxicity and inhibits cell value-added. Meanwhile, the effect of nanofiber membranes on cell migration ability was further evaluated by scratch test. As shown in Fig. 3b–c, the migration rate increased in the 12- and 24-h experimental groups, most significantly in the PVB/PVP group, and gradually decreased with the increase of PMB concentration. Therefore, under the premise of effective infection control, the dosage of polymyxin B should be strictly controlled.

Besides, to assess the in vitro cytotoxicity of nanofibrous excipients on skin cells, we tested the cytotoxic effect of excipients on NIH/3T3 fibroblasts. The live/dead staining of all groups for 24 h is shown in Fig. 3d. Most of the cells in the PVB/PVP, PVB/PVP-PMB1, and PVB/PVP-PMB5 groups survived (green) and showed spindle-like morphology. A small number of cells in the PVB/PVP-PMB10 group died (red), indicating that excess polymyxin B is highly cytotoxic and can lead to cell death. In addition, in vivo, histological analyses further evaluated the effects of localized PMB application via electrospinning on the kidney and nervous system. As shown in Fig. S3, there was no apparent damage to kidney and brain tissues, indicating that the localized application of PMB via electrospinning -loaded PMB had no obvious toxic effects on these organs. Therefore, the electrospinning silk scaffold can be used as a drug delivery device for the local application of PMB.

Overall, the results of dead/living cell staining were consistent with the results of cell proliferation assay and scratch assay, and the dosage of PMB should be strictly controlled to reduce the toxic side effects. Therefore, the nanofibrous and its components are not cytotoxic, have good cytocompatibility, are suitable for in vivo testing, and can be applied as potential wound dressings.

### 2.4 In vitro antimicrobial performance testing

Diabetic wounds are highly susceptible to infection by Gram-negative bacteria due to impaired local immunomodulation and the complex wound microenvironment. Therefore, wound dressings that are effective against Gram-negative bacterial infections are needed. Among them, *Escherichia coli* and *Pseudomonas aeruginosa* are the most common strains of Gram-negative infections in diabetic wounds [6], and *Pseudomonas aeruginosa* is classified by the Centers for Disease Control and Prevention (CDC) as a bacterium that poses a serious clinical threat. This Gram-negative opportunistic pathogen causes various infections in healthcare environments, such as pneumonia and bacteremia, which can be severe enough to lead to patient death. For this reason, this study evaluated the antibacterial activity of nanofibrous dressings against *E. coli* and *Pseudomonas aeruginosa* using the plate counting method. As shown in Fig. 3e, PVB/PVP had no antimicrobial effect and loaded with different concentrations of PMB, had strong antimicrobial effects against Gram-negative bacterial strains (*E. coli* and *Pseudomonas aeruginosa*). Among them, the low attention of PMB was insufficient for antimicrobial activity and preventing the spread of bacterial infections. When PMB was 5 mg/ml, its antimicrobial effect was stronger, effectively preventing bacterial infection spread. It helped reduce inflammation and eliminate necrotic cells in the damaged tissues, thus promoting microorganism-free wound surfaces and accelerating chronic wound repair and regeneration. However, as shown statistically in Fig. 3f, there was no statistically significant difference in the antimicrobial strength between the PVB/PVP-PMB5 group and the PVB/PVP-PMB10 group, suggesting that an overdose does not increase the antimicrobial effect of polymyxin B. Therefore, it is very necessary to control the content of polymyxin B to reduce drug abuse and the production of antimicrobial microorganisms based on effective antimicrobial activity. Thus, the antimicrobial results of electrospinning silk scaffolds piggybacked with PMB suggest that electrospinning silk fiber scaffolds piggybacked with PMB are expected to be a robust antimicrobial strategy against Gram-negative bacterial infections for tissue engineering and biomedical applications.

In conclusion, pure PVB/PVP nanofibrous scaffolds have no antimicrobial effect and do not control infections. The antibacterial effect against Gram-negative bacteria increases gradually with the increase of PMB-loaded content. Still, the antibacterial effect of excessive PMB does not continue to grow, so it is very necessary to control the dosage of PMB. Firstly, PMB can kill pathogenic microorganisms by destroying the bacterial extracellular membrane, effectively preventing Gram-negative bacterial infections and transforming the infected wound into a suitable sterile microenvironment. In addition, PVB/PVP nanofibrous scaffolds equipped with PMB can effectively slow the release of PMB, which can sustain antimicrobial activity. Thus, our strategy leads to a sustained and potent antimicrobial effect on colonized bacterial-infected wound areas to accelerate Healing quickly.

### 2.5 In vivo infected diabetic wound healing

**Fig. 4:**
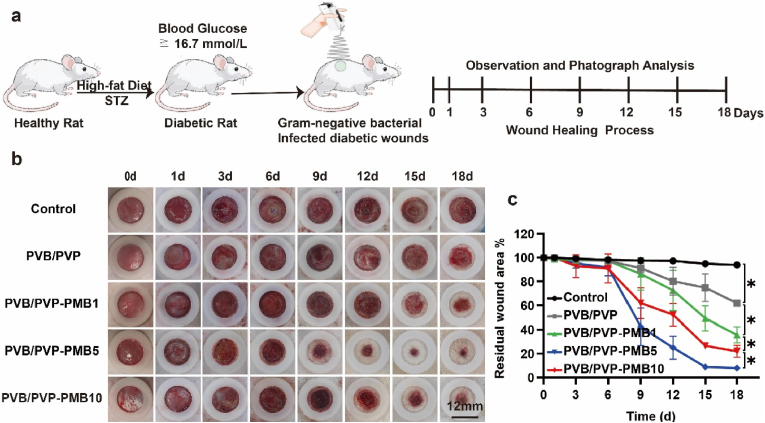
In vivo process of Nanofibrous membranes promoting healing of Gram-negative bacteria-infected type II diabetic wounds. (a) Schematic representation of the construction and treatment of the Gram-negative bacteria-infected type II diabetic wound model. (b) Representative images of the healing process of diabetic wounds treated with nanofibrous membranes at different time points (Scale bar, 12 mm). (c) Statistical results of the residual wound area of diabetic wounds at different time points (n = 3).

In this study, a rat model of Type II Diabetic induced by high-fat diet plus streptozotocin (STZ) and a model of infected diabetic full-thickness skin defects by *Pseudomonas aeruginosa* and *Escherichia coli* were established to evaluate the effect of nanofibrous scaffolds on wound healing in infected Type II Diabetic rat (Fig. 4a).

#### 2.5.1 Healing of diabetic wounds infected with gram-negative bacteria

The wound residual area, one of the most intuitive evaluation indexes of wound healing, was first used to evaluate the difference in repair effect between different groups. As shown in Fig. 4b–c, the optical images of wound appearance and the ratio of wound bed residual area at 0, 1, 3, 6, 9, 12, 15, and 18 days after treatment with PVB/PVP, PVB/PVP-PMB1, PVB/PVP-PMB5, and PVB/PVP-PMB10, respectively. It was clear that no significant healing was observed in the blank control group throughout the treatment period, suggesting that healing diabetic wounds infected with Gram-negative bacteria was difficult. In contrast, the experimental group showed a trend toward rapid wound healing, suggesting that the experimental group was effective in controlling the infection and promoting the Healing of infected diabetic wounds. As shown in Fig. 4b, within six days, PVB/PVP-PMB1 and PVB/PVP-PMB5 showed a slight reduction of the wound. Still, the groups had no statistically significant difference, mainly because the injuries were in the infected-inflammatory phase. From day 3, epithelial regeneration started in the experimental groups, and the scars all tended to shrink. From day six onwards, the healing trend and differences between groups were gradually obvious, and the experimental group showed a trend of rapid Healing (Fig. 4b), which might be related to the early effective antibacterial and extracellular matrix-like nanofibers to promote macrophage polarization and the immune response modulation ability of PMB. The effective control of infection and regulation of local immune response during the inflammatory phase can then control the local inflammatory state and promote the transition from the inflammatory phase to the proliferative phase of the wound, which further confirms that early and effective control of infection can significantly boost the healing of infected wounds during the treatment of infected wounds. As shown in Fig. 4c, at day 18, the wound residual area in the blank control group was approximately 94 %, significantly slowing down the healing rate compared to previously studied non-infected diabetic wounds, indicating that infection can substantially delay the Healing of diabetic wounds. However, the wound residual area ratio of the pure electrospinning PVB/PVP group (≈62 %) was very different (P < 0.05) from that of the PVB/PVP-PMB1 group (≈35.67 %), PVB/PVP-PMB5 (≈8 %), and PVB/PVP-PMB10 (≈22 %), confirming that nanofibrous scaffold dressings promote diabetic wound repair, mainly due to the association with nanofibrous dressings resembling extracellular matrix, which supports cellular value-addition, migration and polarization. Notably, there was a significant difference (P < 0.05) in the wound residual area of the PVB/PVP-PMB10 group (≈22 %) compared with that of the PVB/PVP-PMB5 group (≈8 %), i.e., it indicated that the rate of wound healing was rather slowed down at the concentration of PMB of 10 mg/kg, which was mainly considered that excessive concentration of polymyxin B had a toxic effect on local wound It is primarily considered that the extreme attention of polymyxin B has a poisonous impact on the local wound cells, which is consistent with the results of cellular experiments. In conclusion, from the macroscopic observation, in the appropriate concentration range, polymyxin B can effectively control the local Gram-negative bacterial infections in diabetic wounds and promote the potential for infected diabetic wound healing.

Nanofibrous scaffolds piggybacked with PMB can confer sufficient antimicrobial properties to transform the infected wound into a suitable sterile environment. In contrast, nanofibrous platforms with extracellular matrix-like structures can enhance permeability, absorb excess exudate, accelerate fluid exchange, promote circulation and polarization of immune cells, and reduce inflammatory response to repair infected wounds quickly. Furthermore, in the presence of potent antimicrobial agents, the nano electrospinning scaffolds with extracellular matrix-like structure showed excellent cytocompatibility, appropriate stability, and tunable bioactivity to improve proliferation, cell migration, and transformation functions in the development of diabetic wounds and accelerate the Healing of infected diabetic wounds. Thus, the obtained in vivo results suggest that nanofibrous scaffold-loaded PMB electrospinning fiber mats may be a better fibrous antimicrobial material for promoting early angiogenesis and accelerating the reconstruction of chronically infected wounds in cutaneous diabetic wound healing applications.

#### 2.5.2 Histological examination of diabetic wounds infected with gram-negative bacteria

**Fig. 5:**
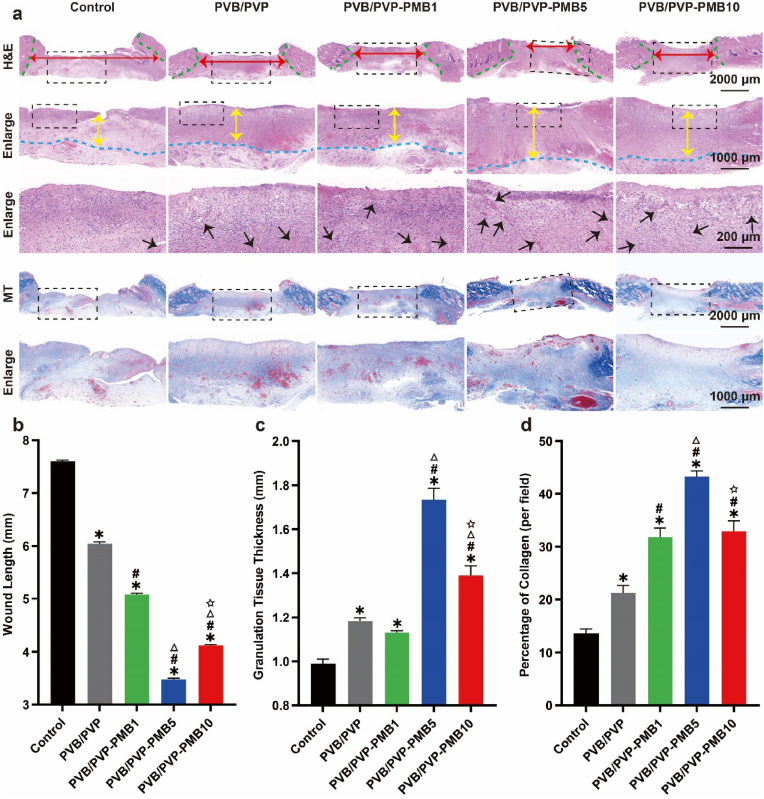
Histological analysis of skin tissues of diabetic wounds treated with nanofibrous membrane on day 18. (a) Representative images of H&E staining (scale bar, 200 μm; scale bar in enlarged figures, 1000 μm, 2000 μm) and Masson trichrome staining (scale bar, 1000 μm; scale bar in enlarged figures, 2000 μm) of regenerated wound tissue. Red double-headed arrows represent the range of wound length. Green dotted lines represent the boundary between the wound and normal tissue. Yellow double-headed arrows represent the range of granulation tissue. Blue dotted lines represent the boundaries of granulation tissue. Black arrows represent vessels. (b) The H&E staining measured the wound length (n = 3). (c) Statistical data of the granulation tissue thickness (n = 3). (d) Statistical data of the percentage of collagen per field (n = 3).

The ability of the prepared nanofibrous scaffolds piggybacked on PMB to repair, regenerate, and heal infected diabetic wounds was assessed by observing the length of the damage, granulation tissue thickness, collagen density, and neovascularization. As shown in Fig. 5, keratin-forming cells migrated to the wound site and formed a new epithelial layer at the wound edge. The regenerated epithelium of the wound in the blank control group was negligible due to the lack of permeability, bioactivity, and antimicrobial activity as the empty control group was not intervened. Compared with the control group, the rate of regenerated epithelium gradually increased in all experimental groups, and the contraction of the wound became more and more obvious. Among them, the regenerated epithelium and wound contraction were most obvious in the PVB/PVP-PMB5 group. The wound healing was optimal, while the regenerated epithelium and wound contraction of the wound in the PVB/PVP-PMB10 group were less than those in the PVB/PVP-PMB5 group, which was the same as that of the macro epithelial image and the curve diagrams, which indicated that the excessive polymyxin inhibited the regeneration of the wound tissue. Due to the permeability of the electrospinning silk scaffolds and the extracellular matrix-like structure that can contribute to cell polarization and the immuno-responsive antimicrobial agent PMB, it provided the required antimicrobial properties in controlling invasive bacterial infections. The electrospinning filaments with extracellular matrix-like properties themselves contribute to local cellular proliferation, migration, and differentiation and eliminate wound exudates from the damaged site, which then provide sufficient bioactive substances to the wound site, which helps to reduce infection and inhibit scar formation at the site of injury. They also enhance fibroblasts and keratinocyte cell proliferation, which accelerates tissue regeneration and modulates local cell differentiation.

Electrospinning filaments and immunoreactive PMB can synergize to have a modulatory effect on local immunity, suggesting that intermolecular interactions between electrospinning filaments and PMB occur early to promote chemotaxis processes. In addition, the length of dermal gaps without appendages could reflect differences in wound repair outcomes. As shown in Fig. 5a–b, the Control group had the largest dermal opening of its length, followed by the PVB/PVP, PVB/PVP-PMB1, PVB/PVP-PMB10 groups, and finally, the PVB/PVP-PMB5 group showed the best repair results, presenting a decreasing and then an increasing trend, indicating that all the nanofibrous dressings could promote wound healing. Still, the reduced healing efficiency in the PVB/PVP-PMB10 group was caused by considering the toxic side effects of excessive polymyxin B. In addition, granulation tissue consists of fibroblasts, other cells, and extracellular matrix. The thickness of the granulation tissue is also an important indicator for assessing the wound healing process. As shown in Fig. 5a–c, it can be observed that fresh granulation tissue (The middle-lightened area) can be seen in all groups, indicating that the wounds are in the proliferative stage of wound healing, among which the PVB/PVP-PMB5 group has the most granulation tissue, which is in the rapid proliferation stage, which indicates that the concentration of polymyxin B is the most efficient in promoting the wound healing in the whole when the attention of polymyxin B is at 5 mg/kg, which can regulate the local immunity and increase the generation of granulation tissue at the same time of effectively controlling the infection. Immunity and increase the production of granulation tissue. On the contrary, the thickness of granulation tissue in the PVB/PVP-PMB10 group was lower than that in the PVB/PVP-PMB5 group, which was mainly because the high concentration of polymyxin B would lead to local necrosis, which was not conducive to the regeneration of wound tissue.

Collagen is a major component of the dermis extracellular matrix (ECM), plays an important role in ECM reorganization and tissue remodeling, and is important for the skin's strength, structure, elasticity, and wound healing. Therefore, collagen content can also be used as an indicator to assess the effectiveness of wound repair. As shown in Fig. 5a–d Masson trichrome (MT) staining (Blue labeled collagen, red labeled keratin or myofibrils), the gradual deepening and then diminishing of the blue color was visible to the naked eye, indicating that the collagen content gradually increased and then decreased (Fig. 5d). This is mainly related to nanofibrous dressings like the extracellular matrix, which can effectively promote collagen deposition. The decrease in the testimony of PVB/PVP-PMB10 is primarily associated with the toxic side effects of PMB. Thus, the significant difference suggests that nanofibrous scaffolds can significantly depend on collagen deposition.

### 2.6 Macrophage polarization, inflammation, and vascular regeneration

#### 2.6.1 Local immune regulation in vivo

**Fig. 6:**
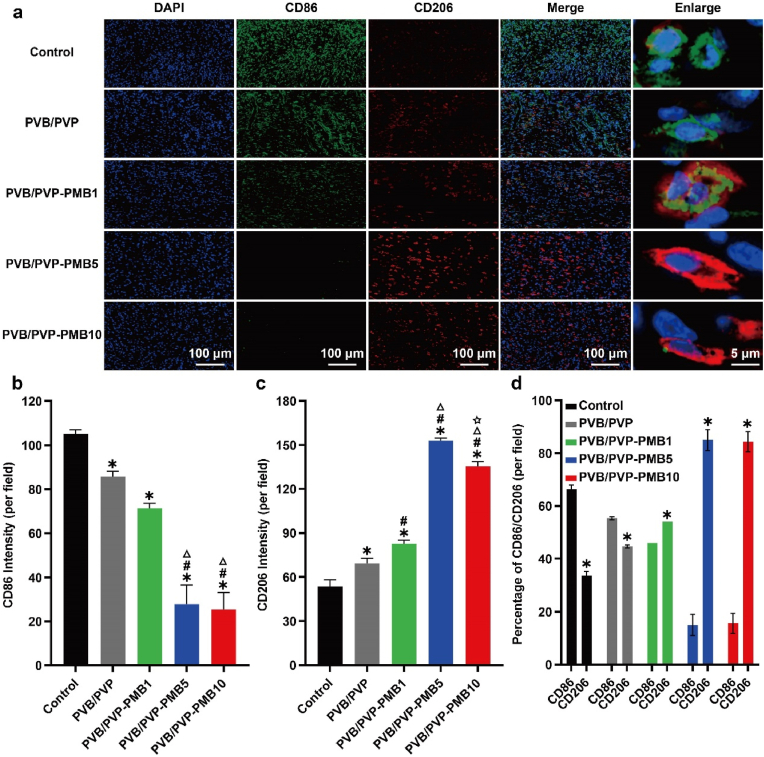
Effect of nanofibrous membranes on local immunity in diabetic wounds. (a) Representative images of immunofluorescence staining of CD86 and CD206. M1 phenotype macrophages (CD86: green), M2 phenotype macrophages (CD206: red), and nucleus (DAPI, blue). Scale bar: 100 μm. Scale bar in enlarged figures: 5 μm. (b) Mean fluorescence density of CD86 (n = 3). (c) Average fluorescence density of CD206 (n = 3). (d) Percentage of CD86 and CD206 (∗P < 0.05 CD206 % vs. CD86 %).

Macrophages play a crucial role in wound healing. M1 macrophages can be involved in pro-inflammatory responses, killing pathogens, and wound debridement, mainly during the first phase of wound healing. Meanwhile, M2 macrophages are associated with anti-inflammatory responses. It contributes to cell proliferation, angiogenesis, and other activities, mainly in the second phase of wound healing [[27], [28], [29]]. During wound healing, macrophages can develop different phenotypes (M1 or M2) in response to environmental stimuli. However, impaired macrophage aggregation, invasion, motility, and phenotypic switching (M1, M2 phenotypic switching) in diabetic wounds led to massive aggregation of M1-type macrophages, persistent chronic inflammation with difficulty regenerating blood vessels, and delayed wound healing [30]. Therefore, modulation of immunity in diabetic wounds is essential to promote diabetic wound healing. In this study, the number of macrophages in vivo and their polarization status M1 and M2 types were characterized by immunofluorescence staining of CD86/CD206, iNOS/Arg-1. CD86/iNOS (Green) as a marker of M1 macrophages and CD206/Arg-1 (Red) as a marker of M2 macrophages. As shown in Fig. 6a and Fig. S4a: M1-type macrophages were highly expressed in the blank group, PVB/PVP group, and PVB/PVP-PMB1, and less in the PVB/PVP-PMB5 and PVB/PVP-PMB10 groups, in contrast to M2 macrophages, which is associated with impaired macrophage phenotypic switching in diabetic wounds. Nano electrospinning silk dressing PVB/PVP with extracellular matrix-like properties modulated local macrophage polarization and promoted macrophage transformation in diabetic wounds. Fewer M1 macrophages and more M2-type macrophages in the PVB/PVP-PMB1 and PVB/PVP-PMB5 groups, with a higher M2 to M1 ratio (Fig. 6b–d and Figs. S4b–d), indicated a reduced inflammatory response and increased anti-inflammatory capacity. It is mainly considered that PMB with immune response ability can effectively regulate the local immunity of diabetic wounds under the synergistic effect of extracellular matrix-like substances and promote the conversion of M1-type macrophages to M2-type macrophages, that is, the wound changes from a pro-inflammatory status to a pro-healing status. The PVB/PVP-PMB5 group showed a significant increase compared with the PVB/PVP-PMB1 group, i.e., the effect of regulating local immunity gradually increased with the increase of PMB concentration. However, surprisingly, the number of M2-type macrophages in the PVB/PVP-PMB10 group did not increase but even decreased compared with the number of M2 macrophages in the PVB/PVP-PMB5 group (Fig. 6b–d and Figs. S4b–d), which was mainly considered that the concentration of excessively high PMB had an obvious toxic effect on macrophages, consistent with previous studies showing that PMB had a killing effect on macrophages. Therefore, the use of nanofibrous dressings to modulate macrophage polarization combined with the immunomodulatory effect of PMB to regulate the immune process in diabetic wounds should be strictly controlled by the content of PMB, and excess will lead to the destruction of macrophages instead, which is not conducive to wound healing.

#### 2.6.2 Inflammatory regulation in vivo

**Fig. 7:**
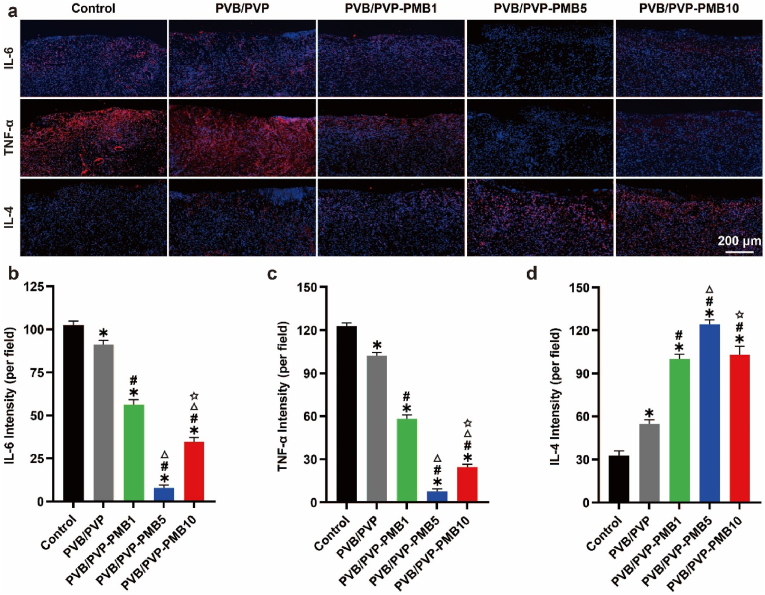
Effect of the nanofibrous membranes on local inflammation in diabetic wounds. (a) Representative images of immunofluorescence staining of IL-6, TNF-α, and IL-4. Scale bar: 200 μm. (b) Average fluorescence density of IL-6 (n = 3). (c) Mean fluorescence density of TNF-α (n = 3). (d) Mean fluorescence density of IL-4 (n = 3).

Impaired immune regulation in diabetic wounds leads to impaired polarization processes in M1-type macrophages like M2-type macrophages, resulting in disturbed inflammation in diabetic wounds [31,32]. The sustained release of inflammatory factors locally in the diabetic damage, which triggers an inflammatory cascade response, leads to massive inflammatory cell infiltration on the wound surface with delayed dissipation, severely affecting diabetic wound healing. Impairment of inflammatory function will lead to failure of antimicrobial control, which in turn may amplify inflammation and complications such as gangrene or sepsis. Thus, the imbalance between pro-inflammatory and anti-inflammatory cytokines is a major feature of impaired wound healing and other complications in diabetic patients, greatly limiting diabetic wound healing. The pro-inflammatory cytokines IL-6, TNF-α, and anti-inflammatory factors IL-4 are associated with inflammatory responses. Therefore, immunohistochemical staining for IL-6, TNF-α, and IL-4 was performed to assess the inflammatory response. As shown in Fig. 7 and Fig. S5-7, pro-inflammatory cytokines (IL-6, TNF-α) gradually decreased and then increased, and anti-inflammatory cytokines (IL-4) gradually increased and then decreased, indicating that as the concentration of PMB increased, the role of immune regulation gradually increased, which led to the effective control of inflammation and a decrease in inflammatory response. However, the toxic side effects of too high a concentration of macrophages inhibited the immunomodulatory effect, the anti-inflammatory ability was reduced, and the inflammatory response increased. In conclusion, piggybacking polymyxin B through electrospinning can effectively regulate the local immunity of diabetic wounds, promote the conversion of M1-type macrophages to M2-type macrophages, and promote the aggregation of M2-type macrophages to avoid long-term inflammation dominated by M1 macrophages leading to the chronic non-healing of diabetic wounds and to effectively control inflammation, improve the local inflammatory microenvironment, and significantly promote the Healing of diabetic wounds.

#### 2.6.3 In vivo evaluation of neovascularization

**Fig. 8:**
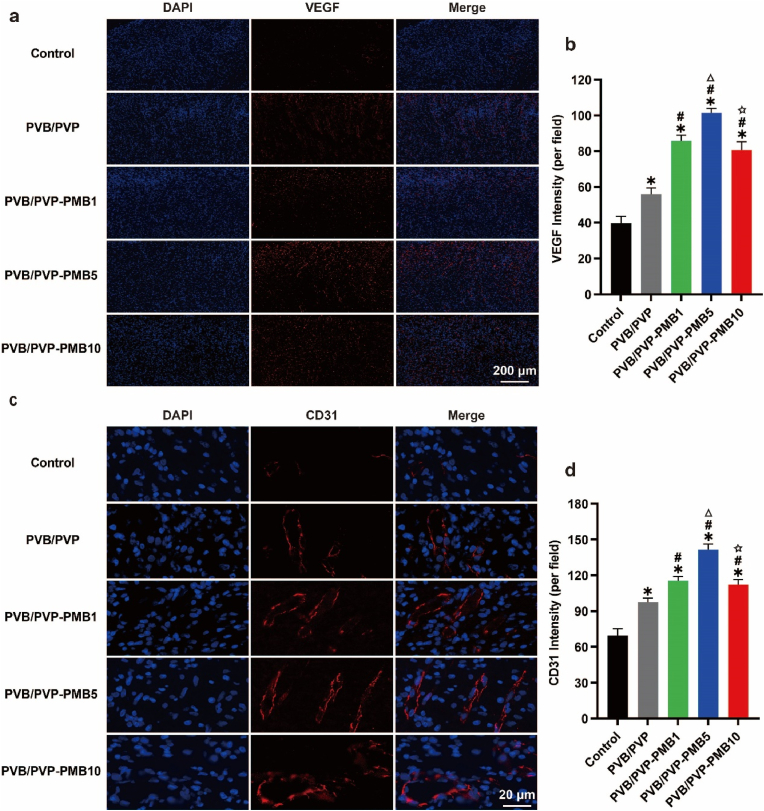
Effect of the nanofibrous membrane on local neovascular regeneration in diabetic wounds. (a) Representative images of immunofluorescence staining of VEGF. Scale bar: 200 μm. (b) Average fluorescence density of VEGF (n = 3). (c) Representative images of immunofluorescence staining of CD31. Scale bar: 20 μm. (d) Average fluorescence density of CD31 (n = 3).

Impaired polarization of M1 and M2 macrophages in diabetic wounds, inflammatory disorders, and bacterial infections are some of the many reasons that can lead to a reduction in neovascularization in diabetic wounds, further making wound healing difficult. However, neovascularization not only brings macrophages and other monocytes into the wound area but also provides necessary substances to the wound site, such as oxygen, nutrients, growth factors, etc., to accelerate wound healing, and therefore, neovascularization is an essential component of the wound healing process. VEGF promotes the regeneration of neovascularization, and consequently, the present study used immunofluorescent staining of VEGF to evaluate the levels of VEGF in each group. VEGF levels. As shown in Fig. 8a–b, with the increase in PMB dose, the VEGF content gradually increased and then decreased, indicating that its role in promoting the regeneration of neovascularization first increased and then decreased. To further investigate the effect of each biological dressing on neovascularization, the quality of neovascularization was assessed by CD31 immunofluorescence staining. As shown in Fig. 8c–d, the numbers of neovascularization in each group first increased and then decreased, consistent with the results of VEGF, indicating that its role in transporting oxygen and nutrients was enhanced and then weakened, so wound healing was reduced in PMB10.

In conclusion, diabetic wound healing can be promoted by an electrospinning silk dressing like the extracellular matrix. Electrospinning silk piggybacked with polymyxin B can not only effectively control Gram-negative bacterial infections but also accelerate diabetic wound healing by regulating local immunity of the wound, promoting the conversion of M1 macrophages to M2 macrophages (The transformation of the wound from pro-inflammatory state to a pro-healing state), controlling the inflammation effectively, and thereby promoting the regeneration of neovascularization.

In vivo experiments showed that PMB could control infection and reduce the adverse effects of endotoxin produced by bacteria on the proliferation and differentiation of local fibroblasts in wounds and wound healing, promoting the Healing of infected diabetic wounds. Among them, PVB/PVP-PMB5 had the best pro-healing effect, mainly considering the antimicrobial impact of PMB and the synergistic immunomodulatory effect between the nanofibrous scaffolds of extracellular matrix-like and PMB with the ability to modulate the immune response. Firstly, PMB at 5 mg/ml can effectively be antimicrobial and transform the wound into a sterile microenvironment. Secondly, the nanofibrous scaffolds with extracellular matrix-like can regulate the polarization process of local macrophages in diabetic wounds. In contrast, PMB can regulate the immune response, and the two synergistically and effectively restrict the regional immunomodulatory power of diabetic wounds (promoting the conversion process from M1 to M2), effectively reducing inflammation, and encouraging neovascular regeneration, significantly accelerating the rapid Healing of diabetic wounds. This means that nanofibrous scaffolds with PMB effectively control infectious diabetic wound infection and promote diabetic wound healing.

## 3 Conclusion

In this study, lightweight and easy-to-use handheld electrospinning in situ synthesized porous nanofibrous scaffolds can be used to cover wounds of any size and irregular shape, and the spun nanoscale electrospinning stands are like extracellular matrices, which can regulate cellular polarization and thus may promote diabetic wound healing. The scaffolds showed excellent porosity, hydrophilicity and water vapor permeability while maintaining hydrophobicity and could locally and sustainably release polymyxin B in infected diabetic wounds, effectively controlling Gram-negative bacterial infections. PVB/PVP and low-dose PMB have good biocompatibility as determined by dead-vital staining and CCK-8. Noteworthy: PMB has an immune response modulating ability, and in combination with the electrospinning of the inner extracellular matrix, it can effectively regulate the local immune function of diabetic wounds and promote the polarization of M2 macrophages, which in turn effectively controls inflammation and promotes vascular regeneration—accelerating rapid Healing of diabetic wounds. Based on the above evidence, we believe that extracellular matrix-like nanofibrous scaffolds based on PMB piggybacking are a reliable therapeutic modality for controlling diabetic wound infections and modulating local immunity in diabetic wounds and can be applied to a wide range of clinical applications.

## 4 Materials and methods

### 4.1 Materials

Polyvinyl butyral (PVB) B75H was purchased from Kuraray (Germany). Polyvinyl pyrrolidone (PVP, 99 %) was purchased from Aladdin (China). Etoh was purchased from GHTECH (China). The polymyxin B (PMB) sulfate and streptozotocin (AR, 99 %, STZ) were purchased from Servicebio Technology Co., Ltd (Wuhan, P. R. China). The NIH/3T3 cells were provided by Solarbio Co., Ltd (Beijing, P. R. China). The primary antibodies of interleukin-6 (IL-6), Tumor necrosis factor-alpha (TNF-α), interleukin-4 (IL-4), Cluster of differentiation 86 (CD86), Cluster of differentiation 206 (CD206), inducible nitric oxide synthase (iNOS), Arginase-1 (Arg-1), Cluster of differentiation 31 (CD31), and Vascular endothelial growth factor (VEGF) were purchased from BIOSS Biotechnology Co., Ltd (Beijing, P. R. China). The Gram-negative *Pseudomonas aeruginosa* and *Escherichia coli* (ATCC 27853 and ATCC 25922) was obtained from the Department of Laboratory Medicine, Daping Hospital (Chongqing, China).

### 4.2 Preparation of precursor spinning solution

Firstly, PVB 3 % (w/v) and PVP 1 % (w/v) were dissolved in EtOH and stirred for 3 h at room temperature to form a mixed solvent. Then, polymyxin B (0 mg/ml, 1 mg/ml, 5 mg/ml, 10 mg/ml) was dispersed in the mixed solvent and stirred for 5 min to form precursor suspension spinning solutions, respectively. And these spinning solutions were named PVB/PVP, PVB/PVP -PMB1, PVB/PVP -PMB5, and PVB/PVP -PMB10, respectively.

### 4.3 In situ electrospinning process

About 5 mL of precursor solution was drawn into a 5 mL syringe with a 23 G metal needle. It was then placed into a portable electrostatic spinning device (HED-01, Qingdao Nuokang Environmental Protection Technology Co., Ltd, Shandong, P. R. China) with a handheld spinneret. The high voltage of the device was about 12 kV fixed voltage. Electrostatic spinning of the nanofibrous membrane was done by pressing the injector with a finger at 10 cm from the wound. The specific spinning process is shown in Scheme.

### 4.4 Properties and characterization of electrospinning nanofibrous

#### 4.4.1 Characterization

The morphology of the electrostatically spun nanofibrous was examined by scanning electron microscopy (SEM, METTLER TOLEDO JSM-6510, Japan). The fiber diameters of the nanofibrous membranes were randomly selected from 100 fibers, which were measured and calculated by ImageJ software. The Fourier transform infrared spectrometer (Thermo Scientific Nicolet iS10) was used to measure the samples' Fourier infrared spectra (FT-IR, range 400–4000 cm^−1^). An optical contact angle (CA) meter (DSA30 KRUSS, Germany) was used to determine the aqueous CA values of the pieces.

#### 4.4.2 Tensile property

The tensile properties of the nanofibrous membrane were tested using a tensile machine (AGS-X, 100 N, SHIMADZU, Japan). The membranes were cut into dumbbell shapes and tested at a constant tensile speed of 100 mm/min before testing.

#### 4.4.3 Water contact angle and water vapor transmission rate

A droplet shape analyzer (DSA30, KRÜSS, Germany) was used to test the contact angle of the self-extracting dressings by dropping 5 μL of water droplets. A water vapor transmission rate tester (WVTR-C3, SYSTESTER, China) was used to test the dressings' water vapor transmission rate (WVTR) according to the ASTM E96 test method.

#### 4.4.4 Drug release

An ultraviolet (UV) spectrophotometer (UV-1780) detected the effect of PMB's drug release effect. The exact specification of 20 mg nanofibrous membrane was taken, and each sample was immersed in 20 mL of PBS buffer solution (pH = 7.2–7.4). The suspension was kept at 37 °C at a constant temperature, and the humidity chamber shook at 80 min. Absorbance was measured by UV-Vi's spectrophotometer.

### 4.5 In vitro cellular biocompatibility

#### 4.5.1 In vitro cell proliferation assay

The effect of nanofibrous membrane dressings on the proliferation and viability of NIH/3T3 fibroblasts was assessed by the cell counting kit-8 (CCK8) to test nanofibrous membranes' biocompatibility. Nanofibrous precursor spinning solutions were sterilized by ultraviolet rays for 30 min, and different electrospinning dressings (PVB/PVP, PVB/PVP-PMB1, PVB/PVP-PMB5, PVB/PVP-PMB10) were prepared by a handheld electrospinning machine. Different electrospinning dressings were co-cultured with phosphate buffered saline (PBS) at 37 °C for 24 h to prepare the extracts of each material. Then, the sections of the tested samples were filtered through sterile disposable filters. And the extracts were diluted to 10.0 mg ml^−1^. The cell culture medium was Dulbecco's Modified Eagle Medium (DMEM) containing 10 % fetal bovine serum (FBS) and 1 % penicillin/streptomycin. Cells were cultured for more than three generations before use to maintain stability. A concentration of 5 × 10^4^/ml NIH/3T3 cell suspension was added to 96-well plates individually (100 μl per well) and pre-cultured in a 5 % CO_2_ incubator at 37 °C for 24 h. The extracts were then added to each well for co-cultivation individually, and 10 μL of CCK solution was added to each well at the 24th and 48th hour, respectively. After 2 h of incubation, the absorbance at 450 nm was measured by a microplate reader, and cell viability was assessed based on the absorbance.

#### 4.5.2 In vitro cell scratch test

The exact number of NIH/3T3 cells were homogeneously inoculated in 6-well plates and co-cultured with extracts of different materials when the cell density reached about 50 %. The cell scratching operation starts when the cell density reaches 90 % or more. Firstly, a 200 μl tip was used to draw a cross in the 6-well plate, and then PBS buffer was used to wash away the scratched cell debris carefully. Then, 2 ml of serum-free DMEM was added to the 6-well plate and incubated in a 5 % CO_2_ cell culture incubator at 37 °C. The cells were removed at 0 h, 12 h and 24 h after incubation, photographed under a microscope and the images were stored. Finally, the area of cell migration was measured using Image J software to compare the differences in the horizontal migration ability of cells in each group.

### 4.6 In vitro cell dead-living staining assay

In this study, NIH/3T3 cells were inoculated at a 5 × 10^4^ ml^−1^ density in 24-well plates for 24 h. Then, the cells were treated with extracts of electrospinning of different materials (PVB/PVP, PVB/PVP-PMB1, PVB/PVP-PMB5, PVB/PVP-PMB10) for 24 h. After treatment, cells were washed with PBS. Dead/living cells were then stained with calcein AM and propidium iodide (PI) fluorescent dyes and incubated for 30 min. Live cells were stained green by calcein AM, and dead cells were stained red by PI. The samples were then observed and photographed under a confocal microscope.

### 4.7 In vitro antimicrobial test

The antimicrobial properties of various electrospinning silk dressings (PVB/PVP, PVB/PVP-PMB1, PVB/PVP-PMB5, PVB/PVP-PMB10) were tested by colony counting. 0.5 ml of various electrospinning silk dressings (PVB/PVP, PVB/PVP-PMB1, PVB/PVP-PMB5, PVB/PVP-PMB10) and PBS (as a blank control group) were added to the 24-well microtiter plates, respectively. 0.5 ml of *Pseudomonas aeruginosa* or *Escherichia coli* (*E. coli*) bacterial suspensions at a concentration of 10^8^ CFU/mL were injected into each well after UV treatment overnight, respectively. After 24 h of incubation at 37 °C, the bacterial solution was aspirated, 100 μL was serially diluted 10^4^ times, and 100 μL of the bacterial solution was spread onto freshly prepared agar plates using a pipette. The plates were incubated at 37 °C for 24 h. The growth of each bacterium in the petri dishes was then observed and photographed, and the number of remaining live colonies of *Pseudomonas aeruginosa* and *Escherichia coli* in the suspension was estimated using colony-forming units (CFU) counting method.

### 4.8 Modelling of type II diabetic rats

Twenty-four Sprague-Dawley male rats were housed in three per cage at 22 ± 2 °C and under light control (12/12 h dark-bright cycle). After one week of adaptive feeding (Water and food were provided at the libitum), they were fed a high-fat diet for six weeks. After 12 h of fasting and water deprivation, a model of Type II Diabetes mellitus (TIIDM) was established by injecting streptozotocin (STZ) (35 mg/kg) through the tail vein (STZ dissolved in 0.1 mol/L sodium citrate buffer at pH 4.5). 3 and 7 days later, the blood glucose levels were measured through the tail vein. The blood glucose over 16.7 mmol/L was identified as TIIDM for further experiments.

Six-week-old SPF-grade Sprague-Dawley male rats weighing 200–220 g were purchased from Liaoning Changsheng Biotechnology Co Ltd (Liaoning, P. R. China). All animal experiments were conducted in a specific pathogen-free environment. All experiments involving rats were approved by the Laboratory Animal Ethics Committee of Jilin University (Changchun, P. R. China, KT202303069) and followed the International Code of Ethics and the National Institutes of Health Guidelines for the Care and Use of Laboratory Animals.

### 4.9 Assessment of in vivo gram-negative bacterial infected diabetic wounds

The effect of nanofibrous dressings on infected wound healing in vivo was assessed using an infected diabetic wound model. The precursor spinning solutions (PVB/PVP, PVB/PVP-PMB1, PVB/PVP-PMB5, PVB/PVP-PMB10) were sterilized by fully exposing them to ultraviolet light for 30 min and preloaded into sterilized syringes. Fifteen diabetic rats were randomly divided into five groups: blank control group, PVB/PVP group, PVB/PVP-PMB1 group, PVB/PVP-PMB5 group, and PVB/PVP-PMB10 group. The animals were fed in a single cage, fed a normal diet, and had free access to water. All surgeries were performed under aseptic conditions. Rats were anesthetized using the inhaled isoflurane (5 %) vapor method. The rats were fixed on the operating table, the diabetic rats were shaved and depilated with a razor and depilatory cream to expose the back fully, and the surgical area was disinfected with alcohol (75 %). Then, a circular full-layer skin defect with a diameter of 1.2 cm was made on the back of rats using a drill, and equal amounts of *Pseudomonas aeruginosa* and *Escherichia coli* (1 × 10^6^ CFU in 200 μL PBS) were inoculated into the wounds of each group of rats respectively to obtain the gram-negative bacterial infected diabetic wound model. The injuries were observed after 24 h, and the wound surfaces crusted over with purulent exudate visible under the scabs. After simple wound debridement, the wound site was immediately covered in situ with a nanofibrous dressing spun by a handheld portable electrospinning machine. The wound aids were then fixed with a non-woven band-aid to keep the wound clean. To mimic the clinic, the scars were gently scrubbed with saline, and cotton swabs every three days for simple debridement (The control wounds were also debrided according to this method), and then the wounds were spun with a handheld portable electrospinning machine to spin nanofibrous on the wounds and fixed in place as described above. Macroscopic images of the wounds in each group were observed and recorded using a digital camera on days 0, 1, 3, 6, 9, 12, 15, and 18, and the wound area was measured using ImageJ to quantify the residual area of the wounds, which was calculated using the following formula.

$$\text{Wound}\;\text{Residual}\;\text{Area}\;\left(\%\right)=\left[{W\;}_{\left(0,1,3,6,9,12,15,18\right)}/W_{\left(0\right)}\;\right]\times 100\%$$

W _(0)_ and W _(0, 1, 3, 6, 9, 12, 15, 18)_ represent the wound exposure areas on days 0, 1, 3, 6, 9, 12,15, and 18.

### 4.10 Histological analysis

On the 18th day, immediately after euthanasia of diabetic rats, full-thickness skin tissue with a diameter of about 12 mm around the incision was taken, fixed with 4 % paraformaldehyde, embedded in paraffin wax, and sliced into 4 μm thick sections. Tissue sections were stained with hematoxylin and eosin (H&E) staining, Masson trichrome (MT) staining for histological analysis, and photographs were taken with a microscope. Quantitative analyses of granulation tissue thickness and collagen content were performed using Image J software. Kidney and brain tissues were also taken for H&E staining to assess the effect of topical application of polymyxin B on the kidney and brain.

### 4.11 Immunofluorescence staining

#### 4.11.1 Immunofluorescence (IF) mono staining

Paraffin sections were dewaxed and rehydrated with xylene and ethanol, incubated with 3 % hydrogen peroxide for 5–10 min at room temperature to seal endogenous peroxidase activity, then boiled in sodium citrate buffer, and antigen recovery was blocked with 5 % bovine serum albumin; incubated for 10 min at room temperature. The following steps were washed three times with PBS for 5 min each between efforts: 1: drop of primary antibody (IL-6, TNF-α, IL-4, CD31, VEGF, Rabbit Anti-mouse, BIOSS) solution, incubated overnight at 4 °C in a wet box; 2: drop of secondary antibody (Goat Anti-rabbit, BIOSS), incubated for 50 min at 37 °C protected from light; 3: depth of 4′,6-diamidino-2-phenylindole (DAPI, BIOSS) staining solution (5 mg/mL), re-staining of cell nuclei, incubated at 37 °C for 10 min; finally, photographs were taken by fluorescence microscope. Quantitative analysis was performed with ImageJ software.

#### 4.11.2 Immunofluorescence double-staining

As described above, paraffin sections were dewaxed, dehydrated, and washed three times with PBS. Tissues were closed with 3 % bovine serum albumin at room temperature for 1 h and then washed thrice with TBST. Between each of the following steps, slides were washed with TBST buffer. ① Drop in primary antibody (CD86, iNOS, Rabbit Anti-rat, BIOSS) and incubate overnight at 4 °C in a wet box. ② Drop in secondary antibody (Goat Anti-rabbit, BIOSS) and incubate at 37 °C for 50min, avoiding light. ③ Drop in primary (CD206, Arg-1; Rabbit Anti-rat, BIOSS) antibody and incubate overnight at 4 °C in a wet box. ④ Drop in secondary antibody and incubate for 50 min ⑤ Drop in DAPI staining solution to re-stain the cell nucleus; incubate for 10 min at room temperature away from light. Observe and collect images under a fluorescent microscope.

### 4.12 Statistical analyses

All results are expressed as mean ± standard deviation (n = 3) and were analyzed using GraphPad Prism software ((Version 9.0, San Diego, California, USA). One-way analysis of variance (ANOVA) was used, and then the Tukey test was performed using Graph Pad Prism software. P-value <0.05 was considered statistically significant (∗P < 0.05 compared with the Control group, ^#^P < 0.05 compared with PVB/PVP group, ^▲^P < 0.05 compared with PVB/PVP-PMB1 group, unless otherwise stated).

## CRediT authorship contribution statement

**Xiaolan Ou:** Writing – review & editing, Writing – original draft, Formal analysis, Data curation, Conceptualization. **Wenlai Guo:** Supervision. **Heng Tian:** Supervision, Formal analysis. **Daojiang Yu:** Supervision, Formal analysis. **Rui Li:** Supervision. **Guanghui Gao:** Writing – review & editing, Supervision, Conceptualization. **Wenrui Qu:** Writing – review & editing, Funding acquisition, Conceptualization.

## Declaration of competing interest

The authors declare no conflict of interest.

## Data Availability

Data will be made available on request.
