# Supplementary material for: Portable direct spraying porous nanofibrous membranes stent-loaded polymyxin B for treating diabetic wounds with difficult-to-heal gram-negative bacterial infections
Source: Mater Today Bio. 2024 Nov 24;29:101365. doi: 10.1016/j.mtbio.2024.101365 (PMC11648811; doi:10.1016/j.mtbio.2024.101365)
Supplement: Multimedia component 1 [file mmc1.docx]

**Portable direct spraying porous nanofibrous membranes stent-loaded polymyxin B for treating diabetic wounds with difficult-to-heal gram-negative bacterial infections**

Xiaolan Ou^1,2^, Wenlai Guo^1^, Heng Tian^1^, Daojiang Yu^2^, Rui Li^1^, Guanghui Gao^3^, and Wenrui Qu^1,4^*^*^*

^1^Department of Hand Surgery, The Second Hospital of Jilin University, Changchun, Jilin Province, 130041, China. [quwenrui@jlu.edu.cn](mailto:quwenrui@jlu.edu.cn) (Wenrui Qu).

^2^Department of Plastic Surgery, Nuclear Industry 416 Hospital, the Second Affiliated Hospital of Chengdu Medical College, Chengdu 610051, China.

^3^Polymeric and Soft Materials Laboratory, Advanced Institute of Materials Science, School of Chemical Engineering, Changchun University of Technology, No. 2055,

Yan’an Street, Changchun 130012, China.

^4^Joint International Research Laboratory of Ageing Active Strategy and Bionic Health in Northeast Asia of Ministry of Education, Jilin University, Changchun, Jilin Province, 130041, China.

1. Results and discussions

**2.1 Handheld electrospinning machine and fabrication of nanofibrous membranes**

**
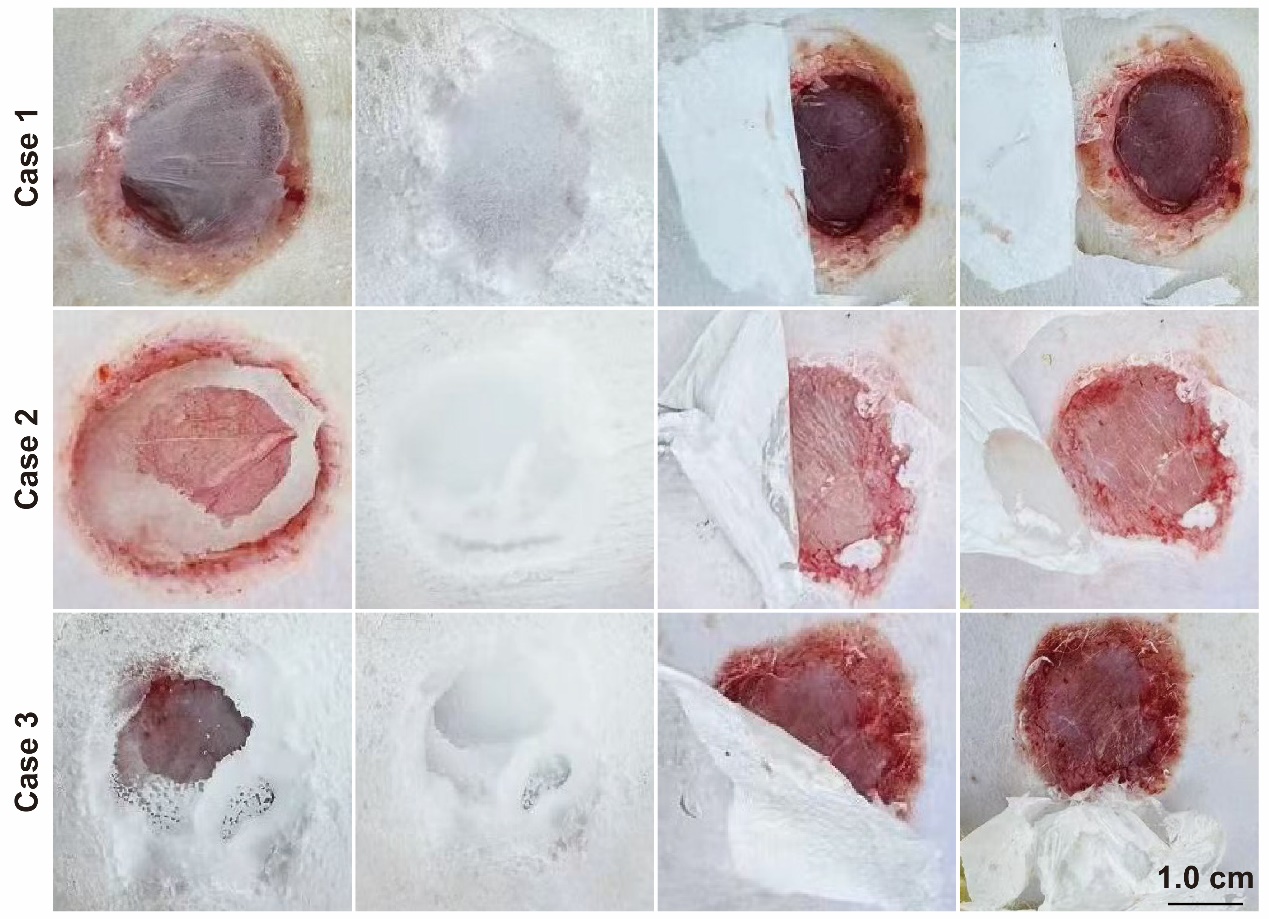
**

**Fig. S1**: Critical steps of in-situ spinning by handheld electrospinning machine to cover the wound and uncover the nanofiber membrane from the wound. Scale bar: 1.0 cm.

- - 1. Water contact angle, porosity, and water vapor transmission rate (WVTR) of nanofibrous membranes


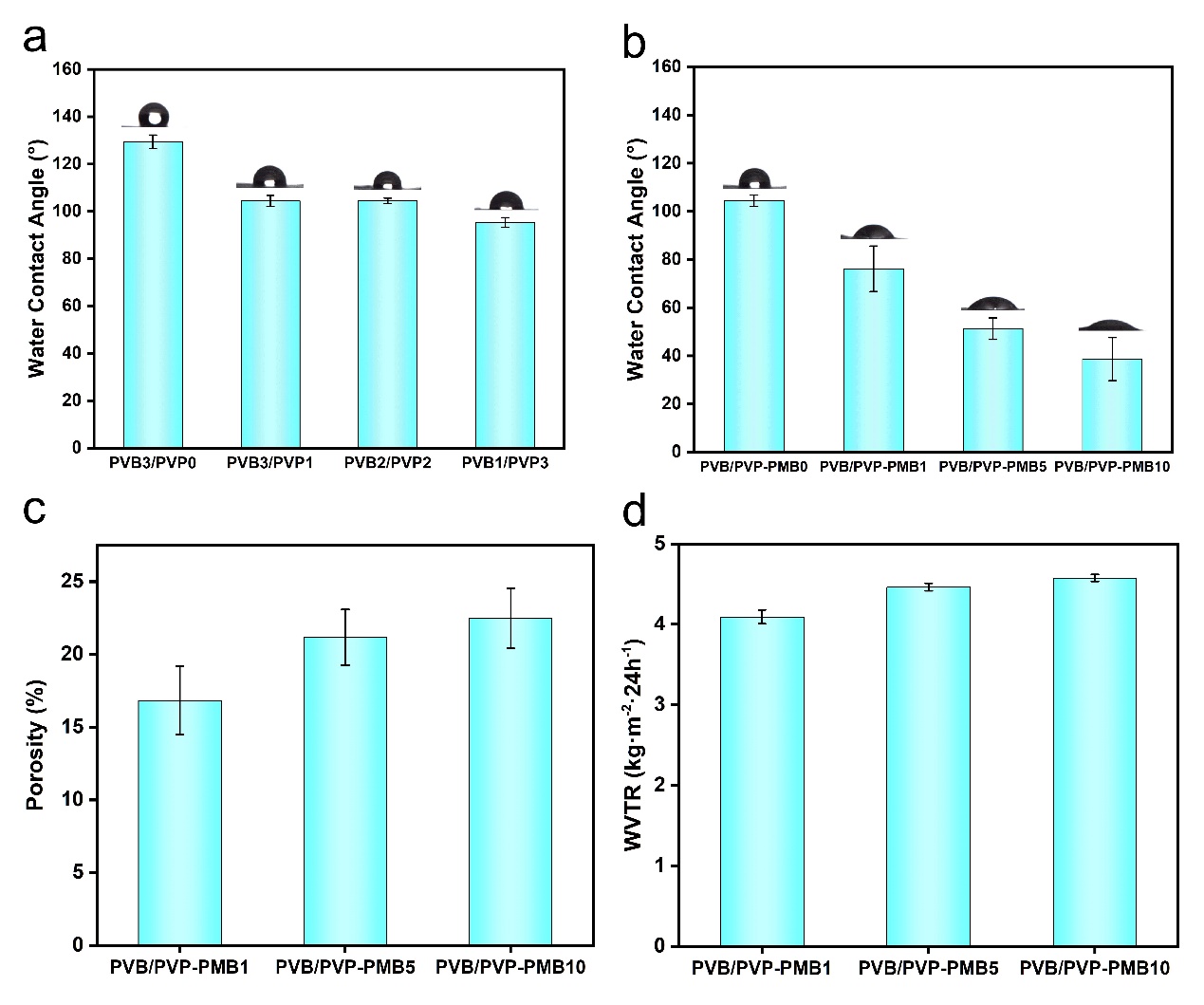


**Fig. S2.** Water contact angle (a, b), porosity (c), and water vapor transmission rate (d) of nanofibrous membranes.

- 1. In vitro and in vivo biocompatibility testing


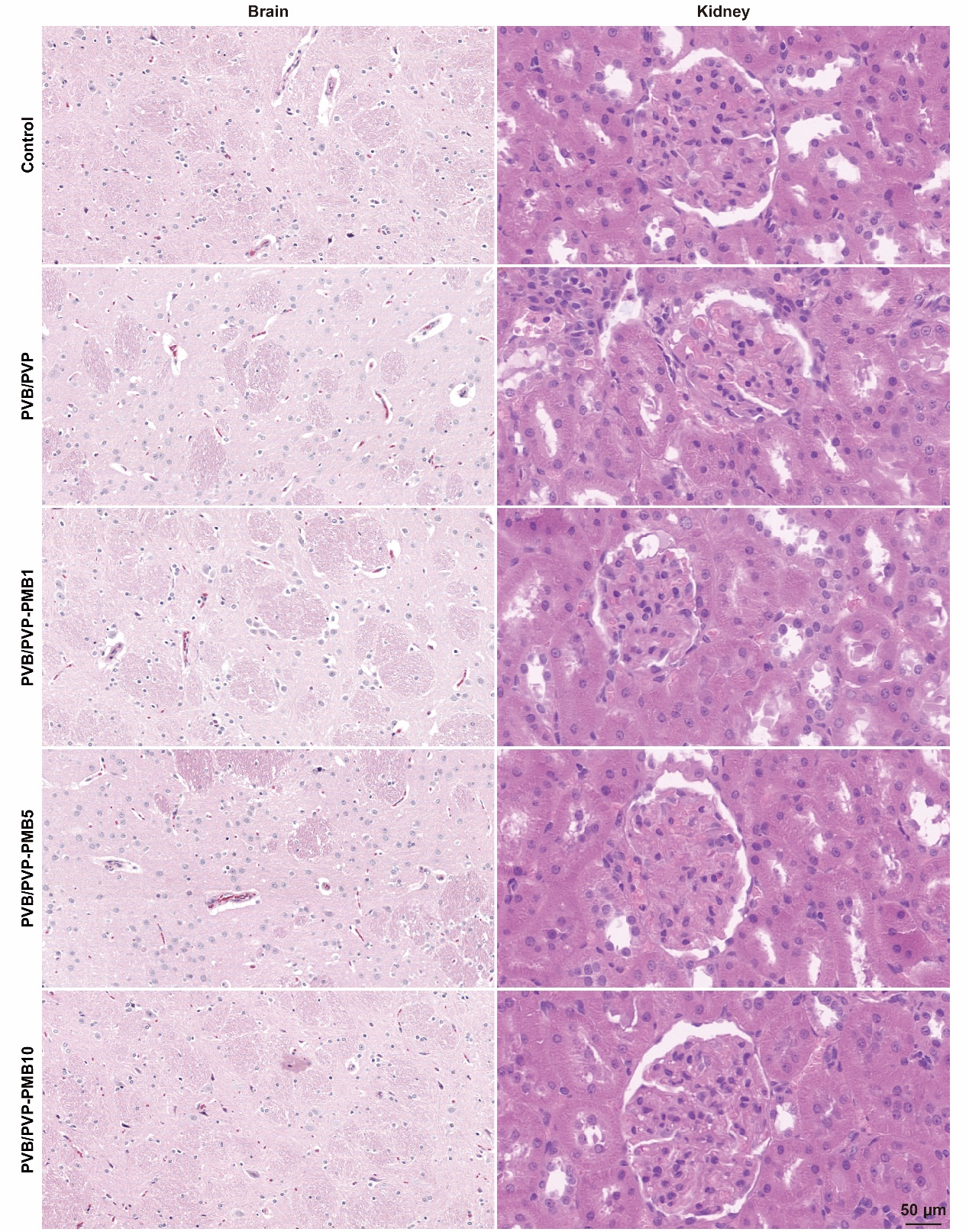


**Fig. S3.** In vivo toxicity: H&E-stained images of Kidney and brain tissues of the rat on day 18. Scale bar, 50 μm.

1. Macrophage polarization, inflammation, and vascular regeneration

2.8.1. Local immunomodulation


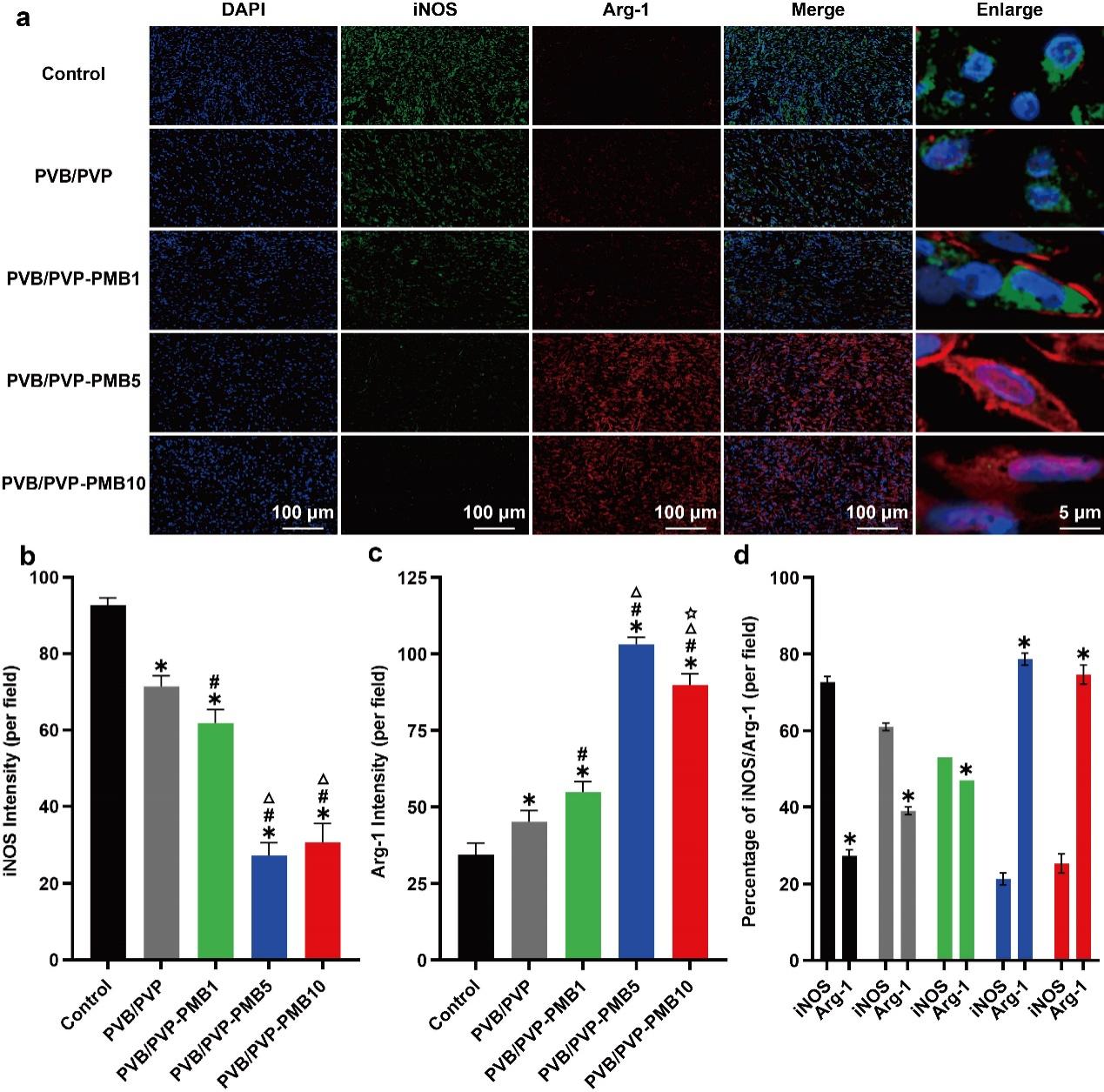


**Fig. S4. Effect** of nanofibrous membranes on local immunity in diabetic wounds. (a) Representative images of immunofluorescence staining of iNOS and Arg-1. M1 phenotype macrophages (iNOS: green), M2 phenotype macrophages (Arg-1: red), and nucleus (DAPI, blue). Scale bar: 100 μm. Scale bar in enlarged figures: 5 μm. (b) Mean fluorescence density of iNOS (n = 3). (c) Average fluorescence density of Arg-1 (n = 3). (d) Percentage of iNOS and Arg-1 (*P < 0.05 Arg-1% vs iNOS %).

2.8.2. Inflammatory Regulation in Vivo


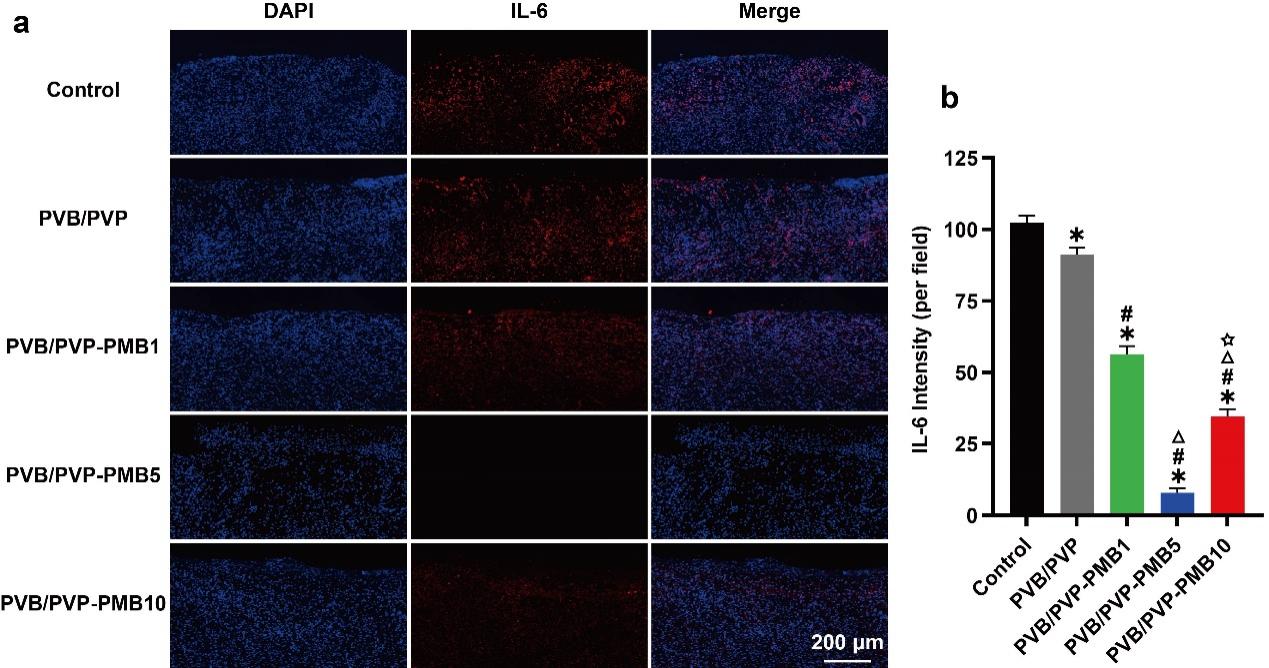


**Fig. S5. Effect of the nanofibrous membranes on local inflammation in diabetic wounds.** (a) Representative images of immunofluorescence staining of IL-6. Scale bar: 200 μm. (b) Average fluorescence density of IL-6 (n = 3).


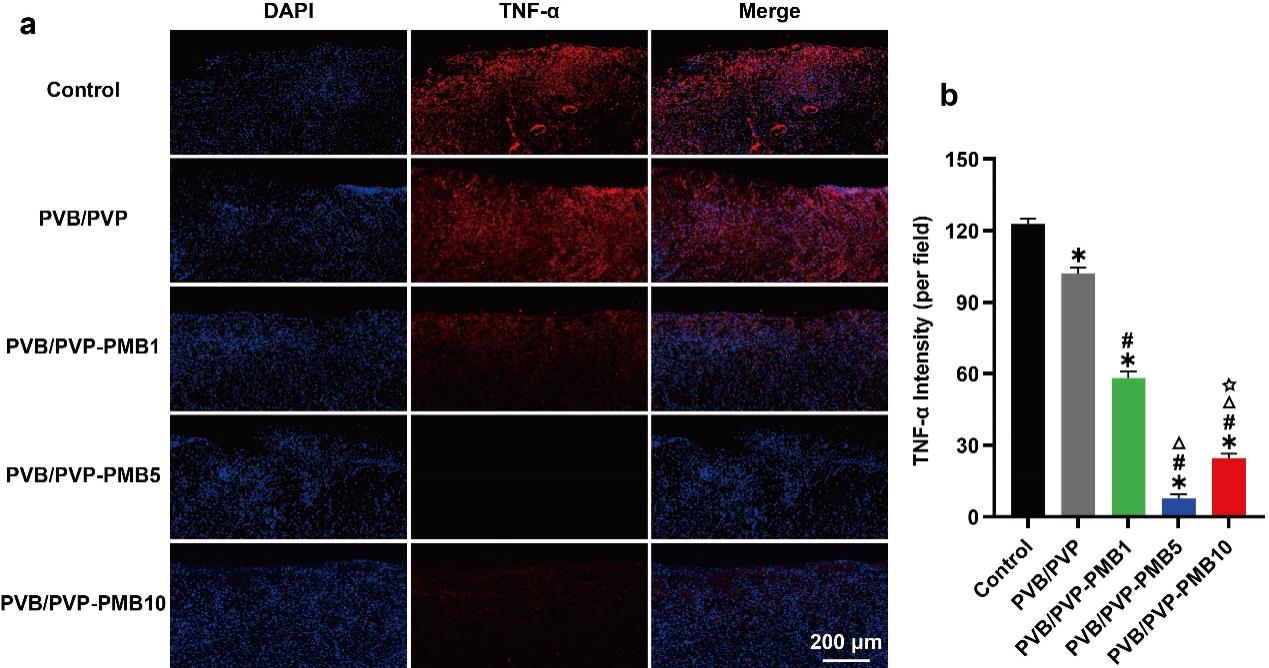


**Fig. S6. Effect of the nanofibrous membranes on local inflammation in diabetic wounds.** (a) Representative images of immunofluorescence staining of TNF-α. Scale bar: 200 μm. (b) Average fluorescence density of TNF-α (n = 3).


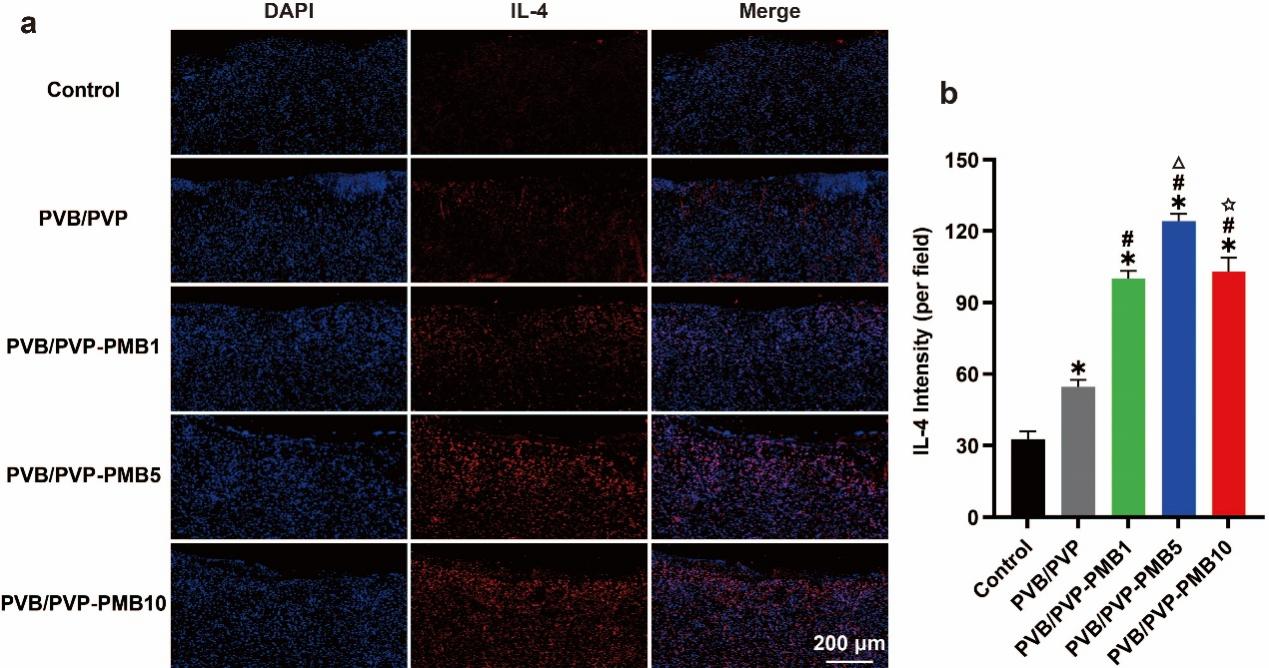


**Fig. S7. Effect of the nanofibrous membranes on local inflammation in diabetic wounds.** (a) Representative images of immunofluorescence staining of IL-4. Scale bar: 200 μm. (b) Average fluorescence density of IL-4 (n = 3).
